# Editorial: Highlights in performance science: music performance anxiety

**DOI:** 10.3389/fpsyg.2023.1328762

**Published:** 2023-11-29

**Authors:** Patrick Gomez, Oscar Casanova, Katarina Habe, Michiko Yoshie

**Affiliations:** ^1^Center for Primary Care and Public Health (Unisanté), Department of Occupational and Environmental Health, University of Lausanne, Lausanne, Switzerland; ^2^Department of Musical, Plastic and Corporal Expression, Faculty of Education, University of Zaragoza, Zaragoza, Spain; ^3^Academy of Music, University of Ljubljana, Ljubljana, Slovenia; ^4^Department of Information Technology and Human Factors, National Institute of Advanced Industrial Science and Technology (AIST), Tsukuba, Japan

**Keywords:** music performance anxiety, stage fright, musician, individual differences, performance science, coping, wellbeing, health

Musical performance activities are the culmination of arduous work and dedication. Musical interpretation is, at its core, an act of openness and vulnerability, which makes stage anxiety a relevant issue in the musical community. Music Performance Anxiety (MPA) is a complex and multifaceted phenomenon, manifesting in different ways and affecting musicians to varying degrees, across all educational levels and musical genres (Yoshie et al., 2009; Casanova et al., 2018; Habe et al., 2019; Guyon et al., 2020).

The Research Topic “*Highlights in performance science: music performance anxiety*” showcases a selection of articles about MPA, authored by leaders in the field. The work presented here highlights the broad diversity of research performed across the Performance Science field and put a spotlight on the main areas of interest. The Research Topic includes 14 original articles written by 41 authors from 11 countries. The articles cover many of the most relevant areas of MPA research, including its conceptualization, phenomenology, assessment, and etiology, as well as individual differences in MPA, ways of managing and coping with MPA, and the consequences of MPA. Three of the 14 contributions are review articles.

Candia et al. showed that heart rate, state anxiety, and errors rated by two experts decreased while calmness increased from the first to the third performance in 18 string players performing three times the same piece in front of different audiences of 15–20 people on the same day. There were no significant changes in self-rated valence (good-bad mood) and energetic arousal (alert-tired). The authors concluded that their findings point to the usefulness of stage training to become accustomed to realistic public self-exposure.

Chang-Arana et al. investigated the effects of pianists' trait MPA, situational stress (rehearsal vs. recital), and familiarity with the piece on listeners' perception and understanding of musical expressiveness. Their preliminary analyses with a group of 30 listeners (10 non-musicians, 10 amateur musicians, 10 semi/professional musicians) showed that perceived expressiveness was significantly affected by pianists' trait MPA and familiarity with the piece, whereas interpersonal accuracy of musical expressiveness was significantly affected by situational stress. These findings were independent of listeners' musical background.

Starting from the observation that the prevalence of MPA has remained largely unchanged over the last 40 years, Herman and Clark reviewed the literature to identify possible reasons for the limited efficacy of current approaches to managing MPA. They synthesize and discuss a broad array of key concepts. They note that MPA is predominantly seen as a negative construct with undesirable symptoms and, accordingly, most interventions aim at managing MPA by ameliorating symptoms. They conclude that depathologizing MPA could open new perspectives and have significant practical and theoretical implications.

Irie et al. analyzed the content of reports by 38 student musicians and semi-structured interviews with eight musicians to find that the experience of mental MPA symptoms started as soon as musicians begin to prepare for public performance, the experience of physiological MPA symptoms peaked shortly before public performance, and the experience of behavioral MPA symptoms peaked during public performance. To deal with these different symptoms, musicians reported to use specific strategies such as positive self-talk, concentration, and deep breathing.

The Kenny Music Performance Anxiety Inventory (K-MPAI) is one of the most widely used questionnaires to assess MPA. In her review article, Kenny, the creator of this instrument, examines the research that has used the K-MPAI and touches on a number of important related topics and constructs such as cross-cultural validation, theoretical and clinical conceptualizations of MPA, depression, low self-esteem, somatization, performance quality, and the idiosyncratic nature of MPA in each musician.

Kirsner et al. take a lifespan perspective on MPA by investigating the potential impact of caregiver experiences and patterns of dysfunctional cognitive schemas during childhood and adolescence on the manifestation and severity of MPA in adulthood. Combining data from a survey with 100 musicians and from interviews with eight musicians, these authors show that high-anxious and low-anxious musicians differed in numerous childhood experiences with their parents and in the development of cognitive schemas related to the themes of failure, catastrophizing, and incompetence/dependence.

Choking under pressure refers to performing worse than expected despite high skills and motivation to perform well. In their study, Lubert et al. investigated the effects of a 10-week psychological chocking intervention comprising acclimatization training, goal setting, imagery, self-talk, and relaxation techniques in six musicians, two dancers and one actress. Their mixed-methods analysis revealed reduction in performance anxiety and fear of negative evaluation, and improved self-efficacy and performance quality.

In their PRISMA-based systematic review article, Mazzarolo et al. explored the strategies that music educators use to help manage their students' MPA and teacher and student perceptions of teachers' role in MPA management. The nine articles included in the review indicate that the most common strategies are simulated performance, positive outlook, preparation, and breathing. Most of these strategies are not specifically employed by the educators to influence MPA; rather, they are part of their regular teaching practice. Most students would like to receive support both from their teachers and experts.

Moral-Bofill et al. investigated the effects of a 12-week electronic intervention program called Self-Regulation Skills for Performing Musicians on flow experience (defined in terms of the six dimensions action-awareness merging, concentration on task, sense of control, loss of self-consciousness, transformation of time, and autotelic experience), MPA and social skills. Compared to a control group, the experimental group reported a significant improvement in flow experience (mainly in sense of control and loss of self-consciousness) and MPA from pre- to post-intervention.

Passarotto et al. investigated the relationship between MPA, practice behaviors (practice time and number of repetitions), and performance quality by monitoring 30 healthy pianists practicing a short musical excerpt. State anxiety correlated positively with practice time and number of repetitions, which the authors interpreted as supporting the hypothesis that more anxious musicians are at higher risk of developing playing-related injuries as a result of overuse and repetitive strain. Pianists who improved their playing were also less anxious in the latter part of the experiment.

Rosset et al. conducted a comprehensive health-related survey among 205 university first-year music students at the beginning of their first semester and 62 students at the end of their second semester. On average, mental health was good at the start of the first year but decreased at the end of the second semester. The article highlights differences between performance majors and music education majors in health-related knowledge and coping abilities and between instrument types in practice time and bodily pain. Moreover, students attending courses on musicians' health improved their knowledge about health risks.

In a sample of 186 university-level classical music students, Sokoli et al. found that students' age, gender, and instrument were significant predictors of their pre-performance affective, cognitive, and somatic experience. The study further found that worsening in performance quality from practice to public performance was reported by almost half of the students and best predicted by pre-performance anxious feelings and breathing-related complaints. The authors suggested that the assessment of MPA could be refined by better taking into account instrument specific performance-related bodily complaints.

Spahn et al. assessed dispositional MPA and state MPA during a concert among 67 young amateur musicians of a brass choir. In line with previous classifications, the authors identified three types of MPA with about 75% of the musicians being assigned to the positive type characterized by low levels of MPA symptoms and high levels of self-efficacy and positive functional coping. The article also provided an analysis of the degree of correlation between dispositional MPA and state MPA.

Yao and Li investigated to what extent the Individual Zone of Optimal Functioning model can help predict performance quality using the three anxiety dimensions somatic anxiety, cognitive anxiety, and self-confidence among 30 college-level piano-major students. The study highlighted the strengths and limitations of this model, the idiosyncratic nature of the relationship between self-perceived anxiety and expert-rated performance quality, as well as important directions for future research in this area.

In conclusion, the articles of this Research Topic greatly contribute to the advancement of our understanding of MPA and its role and implications for musicians' wellbeing, health, and career. They also show avenues and opportunities for further developments, demonstrating that MPA remains a highly relevant topic for research and practice in performance science.

## Author contributions

PG: Writing – original draft. OC: Writing – review & editing. KH: Writing – review & editing. MY: Writing – review & editing.
